# Factors associated with ablation-related thrombus extension following microfoam versus radiofrequency saphenous vein closure

**DOI:** 10.1016/j.jvsv.2024.101815

**Published:** 2024-01-11

**Authors:** Amanda L. Chin, Stephanie D. Talutis, Peter F. Lawrence, Karen Woo, David A. Rigberg, Johnathon C. Rollo, Juan Carlos Jimenez

**Affiliations:** Division of Vascular and Endovascular Surgery, Gonda Venous Center, David Geffen School of Medicine at UCLA, Los Angeles, CA

**Keywords:** Microfoam, Nonthermal, Varicose vein, Thrombus, Saphenous, Ablation

## Abstract

**Objective:**

Polidocanol endovenous microfoam ablation (MFA) is approved by the US Food and Drug Administration for great saphenous vein (GSV) closure, yet there are few published data on the subsequent risk of ablation-related thrombus extension (ARTE). Recent societal practice guidelines recommend against routine postprocedure duplex ultrasound (DU) examination after thermal ablation of the GSV in asymptomatic patients. At present, limited data do not allow this recommendation to extend to MFA. Our aim is to identify characteristics and outcomes associated with ARTE following MFA vs radiofrequency ablation (RFA).

**Methods:**

A retrospective review of a prospectively maintained database was conducted of patients who underwent MFA and RFA closure of incompetent above-knee GSVs. Patients treated for isolated tributary vein treatment or did not have a postprocedure DU examination within 48 to 72 hours were not included. Patients were classified into two groups: ARTE and no ARTE. Demographic data, Clinical, Etiologic, Anatomic and Pathophysiologic class, Venous Clinical Severity Score, operative details, postprocedure (48-72 hours) DU findings, and adverse events were analyzed. Variables that were significant on univariate analysis were evaluated using multivariate logistic regression with the primary outcome being development of ARTE.

**Results:**

Between June 2018 and February 2023, 800 limbs were treated with either MFA (n = 224) or RFA (n = 576). Ninety-six GSVs treated with MFA met the study criteria. One hundred fifty successive GSVs treated with RFA during the same period were included as a comparison group. There was no statistically significant difference in baseline demographics between the two groups. Six patients (2.4%) demonstrated ARTE on postoperative DU examination at 48 to 72 hours (MFA, n = 5 [5.2%]; RFA, n = 1 [0.7%]; *P* = .02). Saphenous vein ablation with MFA (*P* = .045) and a vein diameter of >10 mm (*P* = .017) were associated with ARTE on both univariable and multivariable analysis. All patients who developed ARTE were treated with oral anticoagulants (mean, 15.6 days). Body mass index, Clinical, Etiologic, Anatomic and Pathophysiologic class, Venous Clinical Severity Score, microfoam volume, operative time, and prior deep venous thrombosis were not predictive of ARTE.

**Conclusions:**

ARTE after above-knee GSV closure occurred more frequently after MFA. Our results suggest that a saphenous vein diameter of >10 mm may be associated with ARTE. Despite this finding, all patients with ARTE were treated with short-term anticoagulation with no related complications. Until larger studies with high-risk subgroups have been studied after MFA, DU examination should be performed routinely after this procedure and patients with ARTE anticoagulated until the thrombus retracts caudal to the saphenofemoral junction or is no longer present on DU examination. Current societal guidelines recommending against routine post-thermal ablation DU examination should not be applied to similar patients after saphenous nonthermal MFA ablation.

**Article Highlights**

• **Type of Research:** Single-center retrospective cohort study
• **Key Findings:** Above-knee great saphenous vein closure with microfoam vein diameter >10 mm may be associated with ablation-related thrombus extension after microfoam and radiofrequency closure of 246 limbs in 211 patients.
• **Take Home Message:** Postprocedure duplex ultrasound examination should be performed routinely after microfoam ablation of the above-knee great saphenous vein and patients with ablation-related thrombus extension anticoagulated until the thrombus resolves.

Extension of thrombus into the adjacent deep vein after thermal saphenous vein ablation is known as endovenous heat-induced thrombus (EHIT). Extensive high-quality evidence has demonstrated that EHIT is a low frequency event after thermal great saphenous vein (GSV) ablation with an incidence of approximately 1%.^1^, ^2^, ^3^ Owing to its low rate of occurrence, the 2023 clinical practice guidelines from the Society for Vascular Surgery (SVS), the American Venous Forum (AVF), and the American Vein and Lymphatic Society (AVLS) do not support performance of routine postablation duplex ultrasound (DU) screening in asymptomatic average-risk patients.^4^

The term EHIT does not apply to contiguous deep venous extension of thrombus following nonthermal treatments (ie, microfoam ablation [MFA], cyanoacrylate glue, mechanochemical ablation). Revised nomenclature, ablation-related thrombus extension (ARTE) has been recommended by the same societal guidelines to include these alternate modalities.^4^ Because nonthermal techniques have been introduced more recently, the incidence and risk factors associated with ARTE have not been characterized fully with these newer treatments. The aim of our analysis was to compare the risk of ARTE after primary closure of the above-knee (AK) GSV for MFA vs radiofrequency ablation (RFA).

## Methods

We reviewed a prospectively maintained patient database from procedures in our ambulatory procedure unit. Approval was obtained from our institutional review board for the execution and publication of this study; the requirement for patient consent for participation in the study was waived by our institutional review board based on the policy for minimal risk research. Inclusion criteria was treatment of the AK GSV reflux for symptomatic reflux using RFA or MFA. Limbs that underwent concomitant treatment of superficial tributary veins with MFA (tributary vein sclerotherapy) and RFA (stab phlebectomy) were included. Limbs treated solely for superficial tributary vein reflux were excluded. Patients who were classified as Clinical, Etiologic, Anatomic and Pathophysiologic clinical class 0 and 1 and those who did not undergo at least one postprocedure ultrasound examination (48-72 hours) and follow-up visit were also not included.

Symptomatic patients included with functional limitation and physical findings of chronic venous disease. These signs and symptoms include pain, aching, heaviness, fatigue, itching, edema, lipodermatosclerosis, and both active and healed ulcers. DU confirmation of enlarged saphenous veins of ≥3.5 mm and incompetence of ≥0.5 seconds while standing was required for study inclusion. Before vein closure, documentation of failed conservative therapy with ≥6 weeks of compression therapy (≥20-30 mm Hg). All ulcerated limbs underwent wound care before vein ablation and were compliant with weekly dressing changes, debridement, offloading, nutritional counseling, and multilayer compression.

Our techniques for truncal MFA and RFA have been previously published.^5^^,^^6^ The commercial microfoam used was the standard 1% concentration. All patients in this study had a postoperative DU examination (48-72 hours) performed to assess for target vein closure, ARTE, and deep venous thrombosis (DVT). Postprocedure instruction included the use of 20 to 30 mm Hg thigh-high compression stockings for 2 weeks after vein ablation. Consequent evaluation was performed by a venous specialist 3 to 6 weeks after ablation. After the initial DU examination, we do not perform routine surveillance imaging for asymptomatic patients.

Technical success (closure) was defined as lack of truncal vein flow within 5 cm from the saphenofemoral junction (SFJ). Nonclosure was defined as persistent venous flow present for >5 cm from the SFJ. Patients were then stratified into two groups: ARTE and no ARTE. Preprocedureal variables were evaluated between the two groups. These included demographics, body mass index, truncal vein diameters, prior DVT, the use of oral anticoagulants at the time of ablation, the presence of deep venous incompetence, reflux times and Clinical, Etiologic, Anatomic and Pathophysiologic Clinical Class and Venous Clinical Severity Scores (VCSS). Operative variables included GSV diameter, volume of microfoam used, tributary vein sclerotherapy, tributary vein phlebectomy, and procedure times. Postprocedure variables included vein closure, improvement of symptoms, VCSS, complete ulcer healing rates, ARTE, superficial thrombophlebitis, and DVT.

### Statistical analyses

Differences between the ARTE and no ARTE group were evaluated using the χ^2^ test for categorical variables and the Student *t* test or Kruskal-Wallis test as appropriate for continuous variables. Significance was defined as a *P* value of <.05. Variables that were significant on univariate analysis were evaluated using a multivariate logistic regression model. These included limbs that underwent MFA and with a vein diameter of >10 mm. To determine a size threshold at which the risk of ARTE may be increased, vein diameter was dichotomized at 10 mm; 10 mm is the 75th percentile of vein diameter in the total sample and vein diameters of >10 mm may represent potential outliers in the sample. Statistical analysis was performed using SAS 9.4 (SAS Institute, Cary, NC).

## Results

Eight hundred limbs underwent microfoam (n = 224) and radiofrequency (n = 576) closure during the study period. In the MFA group, 96 limbs (in 76 patients) met the inclusion criteria and were treated for symptomatic reflux of the AK GSV. All of these limbs were included. Of the 224 MFA limbs, 128 were excluded because they were either anterior accessory saphenous veins (n = 17) or below-knee truncal veins (n = 111). One hundred fifty consecutive limbs (in 135 patients) underwent AK GSV RFA and were included as a comparison group.

Preoperative variables stratified by ARTE are reported in Table I. The mean preoperative VCSS for the entire study population was 9.8 ± 3.1. There was no difference in preprocedural VCSS between the two groups. Mean age (*P* = .79), body mass index (*P* = .55), female sex (*P* = .58), history of hypercoagulability (*P* = .81), the presence of deep venous incompetence (*P* = .65), prior DVT (*P* = .90), and reflux times (*P* = .38) were not statistically different between the ARTE and no ARTE groups.

**Table I:** Preoperative demographics stratified by ablation-related thrombus extension (*ARTE*)

	No ARTE (n = 240)	ARTE (n = 6)	Overall (n = 246)	*P* value
Age, years	62.9 ± 14.1	64.3 ± 13.5	62.9 ± 14.1	.79
BMI	29.3 ± 6.3	27.9 ± 7.9	29.2 ± 6.3	.55
BMI ≥30	37.3	37.5	37.3	.99
Female	67.0	50.0	66.6	.58
History of hypercoagulability	0.7	0.0	0.7	.81
Deep reflux	67.4	75.0	67.6	.65
History of DVT	11.1	12.5	11.1	.90
On AC preoperatively	12.5	0.0	12.2	.29
CEAP classification				
C2	33.3	37.5	33.4	.81
C3	29.4	12.5	28.9	.30
C4	17.9	37.5	18.5	.16
C5	7.2	12.5	7.3	.57
C6	11.5	0.0	11.2	.31
Preoperative VCSS	9.8 ± 3.1	9.5 ± 1.9	9.8 ± 3.1	.80
Reflux time	3.5 ± 1.1	3.9 ± 1.2	3.5 ± 1.1	.38

*BMI*, Body mass index; *CEAP*, Clinical, Etiologic, Anatomic and Pathophysiologic; *DVT*, deep venous thrombosis; *VCSS*, Venous Clinical Severity Score.

Values are mean ± standard deviation or percent.

Operative variables stratified by ARTE are reported in Table II. A truncal vein diameter of >10 mm was present in f limbs in the ARTE group (67%) and 47 limbs in the no ARTE group (19.7%). This difference was significant (*P* = .037). The mean vein diameter in the ARTE group was 10.0 ± 2.0 mm compared with the no ARTE group (8.7 ± 3.3 mm) (*P* = .25). There were no significant differences in other procedural variables, including concomitant treatment of tributary veins (*P* = .24), mean microfoam volume used (*P* = .50), or operative times (*P* = .12).

**Table II:** Operative variables stratified by ablation-related thrombus extension (*ARTE*)

	No ARTE (n = 240)	ARTE (n = 6)	Overall (n = 246)	*P* value
Vein diameter, mm	8.6 (6.5, 10.0)	10.4 (8, 12)	8.6 (6.7, 10)	.049
Vein diameter >10 mm	19.7	67.0	20.6	**.037**
Tributaries treated	14.7	0.0	14.3	.24
Maximum tributary diameter, mm	6.9 ± 2.6	n/a	6.9 ± 2.6	n/a
Mean foam volume, mL	6.3 ± 2.7	5.5 ± 1.4	6.2 ± 2.7	.50
Operative time	41.5 ± 21.1	34.6± 9.9	41.2 ± 20.9	.12

*n/a*, Not applicable.

Values are median (interquartile range), percent, or mean ± standard deviation.

Boldface entries indicate statistical significance.

A total of six limbs developed postprocedure ARTE (MFA, n = 5; RFA, n = 1). Saphenous vein ablation with MFA was significantly associated with the development of ARTE (*P* = .045). Details can be found in Table III. Four were class III and two were class II as defined per the current SVS/AVF classification for thrombus propagation into the adjacent deep vein.^7^ All were symptom free and treated with apixaban 5 mg by mouth twice daily. They underwent weekly ultrasound examination until retraction of the clot caudal to the SFJ was noted. The mean duration of anticoagulation (time to retraction caudal to the SFJ or nonvisualization by DU examination) for ARTE was 15.3 days. Two patients in the MFA group developed asymptomatic remote DVTs. The locations included a paired femoral vein (n = 1) and an anterior tibial vein (n = 1). These DVTs were not included in the ARTE group because they were not contiguous with the treated GSV. These patients were treated with the same anticoagulation protocol for a mean duration of 58 days. No embolic or adverse neurological events occurred during the study.

**Table III:** Characteristics for patients with ablation-related thrombus extension (*ARTE*)

Patient	Extent: ARTE class	Vein diameter, mm	Volume of microfoam, mL	BMI	History of prior DVT	Duration of anticoagulation, days	Resolved with anticoagulation?
MFA							
A	II	8	7	17.2	No	8	Yes
B	III	7.5	6	22.4	No	26	Yes
C	III	11.3	6	22.3	No	15	Yes
D	III	12	6	27.6	No	7	Yes
E	II	12	5	33.4	No	22	Yes
RFA							
F	III	12		42.9	Yes	7	Yes

*BMI*, Body mass index; *DVT*, deep venous thrombosis; *MFA*, microfoam ablation; *RFA*, radiofrequency ablation.

The median follow-up was 40.5 days (range, 10-808 days) in the ARTE group and 61 days (range, 3-940 days) in no ARTE. The overall median follow-up was 59 days (range, 3-940 days). The complete closure rates were 100% in the ARTE group and 95.3% in no ARTE (*P* = .82). Improvement of symptoms was reported in 100% and 91.4% in the ARTE and no ARTE groups, respectively (*P* = .39). Overall symptomatic relief was reported by 91.6% of patients. There was no difference between overall mean change in VCSS after GSV closure (ARTE, 2.1 ± 1.1 vs no ARTE, 2.0 ± 1.2; *P* = .44). Reported postprocedure pain (ARTE, 9.7% vs no ARTE, 12.5%; *P* = .79) and superficial venous thrombosis (ARTE, 11.5% vs no ARTE, 12.5%; *P* = .93) were also similar between the two groups. The rate of venous ulcer healing for the study population was 78.1% and all the ulcer patients were present in the no ARTE group.

Significant variables on univariate analysis (GSV closure with MFA and vein diameter of >10 mm) were analyzed using multivariable logistic regression with the primary outcome being development of ARTE. A dichotomous variable of vein diameter of >10 mm vs ≤10 mm was added to the model. On multivariate modeling, both treatment with MFA (*P* = .045) and a vein diameter of >10 mm (*P* = .017) were significant.

## Discussion

Shortly after the advent of thermal saphenous vein ablation two decades ago, early reports of thrombus extension into the adjacent deep venous system suggested that this complication was clinically significant and that early postprocedure surveillance and selective anticoagulation were required to ensure patient safety.^8^ With better patient selection, advancement of technology, and improvement of thermal techniques, the contemporary peer reviewed literature demonstrates a much lower incidence (1%) than described in these early studies. Clinical practice guidelines recently published by the SVS, AVF, and AVLS now recommend against routine ultrasound screening after thermal ablation in normal-risk patients without symptoms.^4^

Because the incidence and clinical risk factors associated with ARTE after newer nonthermal saphenous closure techniques have not been characterized completely, the SVS/AVF/AVLS guidelines do not extend the same recommendation to patients after MFA.^4^ The guidelines recommend, “In an average-risk patient who is asymptomatic following nonthermal ablation of the saphenous vein, routine early postprocedural DUS may be performed to detect ablation-related thrombus extension (ARTE) or DVT.”

In our study, the use of MFA for primary closure of the above knee GSV increased the odds of ARTE by more than nine-fold compared with RFA. In the initial randomized trials (MFA vs placebo) leading to US Food and Drug Administration approval of commercially manufactured microfoam, the incidence of ARTE was reported between 3.9% and 5.5%.^9^, ^10^, ^11^ When ARTEs and DVTs were combined in these studies, the incidence of adverse thrombotic complications overall was between 8.8% and 13.6%.^10^, ^11^, ^12^ Our rates of ARTE were similar; however, our incidence of DVT and overall adverse thrombotic complications were lower (Table IV).

**Table IV:** Comparison of adverse thrombotic events (*ATEs*) after microfoam ablation (*MFA*) across studies

Study	ARTE	Other DVT	Percentage of total ATEs in study	Symptomatic	Patients with ATEs anticoagulated
VANISH-1	5.5	3.3	8.8	22	Not stated
VANISH-2	3.9	6.6	10.5	33	50
Gibson et al	4.1	9.5	13.6	None	20
Chin, et al (current study- MFA Group only)	5.2	2.1	7.3	None	100

*DVT*, Deep venous thrombosis.

Values are percent.

A GSV diameter of >10 mm was also independently associated with the development of ARTE in our analysis. Although increased saphenous vein diameters have been associated with increased risk of EHIT, similar findings have not been reported following MFA.^12^ Varithena (Boston Scientific, Marlborough, MA) is approved by the US Food and Drug Administration for treatment of incompetent GSVs, accessory saphenous veins, and visible varicosities of the GSV system above and below the knee. The current instructions for use do not specify parameters for vein diameters being treated. The Ventricular Tachycardia Antiarrhythmics or Ablation in Structural Heart Disease 2 (VANISH-2) trial treated GSVs between 3.1 and 19.4 mm, but no correlation was reported between ARTE and vein diameter.^11^

The results of our study suggest that, for primary closure of the proximal GSV in the thigh, RFA may result in a lower incidence of ARTE compared with MFA. Our experience has demonstrated that MFA can be used safely in patients with the surveillance and selective anticoagulation protocol described. Saphenous vein closure with MFA also resulted in excellent symptomatic relief and complete ulcer healing rates in this cohort. Despite the increased incidence of ARTE after MFA, no patient experienced thrombus progression or further complications after short-term oral anticoagulation with apixaban. MFA may be preferred compared with RFA in certain clinical situations for the AK GSV. These circumstances include patients with tortuous veins and those who cannot tolerate the multiple tumescent injections required during RFA. Although skin burns occur rarely owing to the use of well-performed tumescence, patients with superficial GSVs close to the skin may also benefit from MFA.

To date, no randomized studies comparing MFA and RFA for GSV treatment have been published. Until larger level 1 comparative studies with high-risk subgroups have been published, DU examination should be performed routinely postprocedure to follow patients with MFA and patients with ARTE anticoagulated until the thrombus retracts or resolves. The low incidence of ARTE after RFA in our study support the most recent SVS/AVF/AVLS guidelines that routine postprocedure DU examination may no longer be required after RFA ablation of the GSV.

Owing to our postprocedural DU surveillance protocol, long-term vein closure durability, symptomatic relief, and late thrombotic events cannot be determined from this analysis. Selection bias may also be present owing to its nonrandomized, retrospective design. Because ARTE was a relatively infrequent event, this factor increases the risk of statistical error. The relatively sample size also limits our ability to conclude definitively that the significance of 10 mm is generalizable to the wider population.

## Conclusions

ARTE after closure of the AK GSV occurred more frequently after MFA than RFA. Our results suggest that a saphenous vein diameter of >10 mm may be associated with ARTE. All patients with ARTE were treated with short-term anticoagulation with no clinical sequelae. Excellent symptomatic relief was reported in both groups. DU examination should be performed routinely after the procedure in both symptomatic and asymptomatic patients and ARTE should be treated with anticoagulation until the thrombus resolves. The most recent SVS/AVF/AVLS clinical practice guidelines recommending against routine post-thermal ablation DU examination should not be applied to similar patients after nonthermal MFA ablation of the AK GSV.

## Author Contributions

Conception and design: KW JJ

Analysis and interpretation: AC ST, PL, KW, DR, JR, JJ

Data collection: AC ST, JJ

Writing the article: AC ST, PL, KW, DR, JR, JJ

Critical revision of the article: AC ST, PL, KW, DR, JR, JJ

Final approval of the article: AC ST, PL, KW, DR, JR, JJ

Statistical analysis: AC ST, KW, JJ

Obtained funding: Not applicable

Overall responsibility: JJ

## Disclosures

J.C.J. and J.R. are consultants and speakers for Boston Scientific and Medtronic.
